# Novel 18-gene signature for predicting relapse in ER-positive, HER2-negative breast cancer

**DOI:** 10.1186/s13058-018-1040-9

**Published:** 2018-09-04

**Authors:** Richard Buus, Belinda Yeo, Adam R. Brentnall, Marie Klintman, Maggie Chon U. Cheang, Komel Khabra, Ivana Sestak, Qiong Gao, Jack Cuzick, Mitch Dowsett

**Affiliations:** 1 The Breast Cancer Now Toby Robins Research Centre at the Institute of Cancer Research, 237 Fulham Road, London, SW3 6JB UK; 2 Ralph Lauren Centre for Breast Cancer Research, Royal Marsden Hospital, London, UK; 3 Olivia Newton-John Cancer Research Institute, Melbourne, Australia; 4 Austin Health, Melbourne, Australia; 5 Centre for Cancer Prevention, Wolfson Institute of Preventive Medicine, Queen Mary University of London, London, UK; 6 Lund University, Skane University Hospital, Faculty of Medicine, Department of Clinical Sciences Lund, Oncology and Pathology, Lund, Sweden; 7 Clinical Trials and Statistic Unit, The Institute of Cancer Research, London, UK; 8 Research Data Management and Statistics Unit, Royal Marsden Hospital, London, UK

**Keywords:** Breast cancer, Oestrogen receptor, Prognostic tests, Biomarkers, Late recurrence

## Abstract

**Background:**

Several prognostic signatures for early oestrogen receptor-positive (ER+) breast cancer have been established with a 10-year follow-up. We tested the hypothesis that signatures optimised for 0–5-year and 5–10-year follow-up separately are more prognostic than a single signature optimised for 10 years.

**Methods:**

Genes previously identified as prognostic or associated with endocrine resistance were tested in publicly available microarray data set using Cox regression of 747 ER+/HER2− samples from post-menopausal patients treated with 5 years of endocrine therapy. RNA expression of the selected genes was assayed in primary ER+/HER2− tumours from 948 post-menopausal patients treated with 5 years of anastrozole or tamoxifen in the TransATAC cohort. Prognostic signatures for 0–10, 0–5 and 5–10 years were derived using a penalised Cox regression (elastic net). Signature comparison was performed with likelihood ratio statistics. Validation was done by a case-control (POLAR) study in 422 samples derived from a cohort of 1449.

**Results:**

Ninety-three genes were selected by the modelling of microarray data; 63 of these were significantly prognostic in TransATAC, most similarly across each time period. Contrary to our hypothesis, the derived early and late signatures were not significantly more prognostic than the 18-gene 10-year signature. The 18-gene 10-year signature was internally validated in the TransATAC validation set, showing prognostic information similar to that of Oncotype DX Recurrence Score, PAM50 risk of recurrence score, Breast Cancer Index and IHC4 (score based on four IHC markers), as well as in the external POLAR case-control set.

**Conclusions:**

The derived 10-year signature predicts risk of metastasis in patients with ER+/HER2− breast cancer similar to commercial signatures. The hypothesis that early and late prognostic signatures are significantly more informative than a single signature was rejected.

**Electronic supplementary material:**

The online version of this article (10.1186/s13058-018-1040-9) contains supplementary material, which is available to authorized users.

## Background

Five years of adjuvant endocrine therapy is standard treatment for patients with primary oestrogen receptor-positive (ER+) breast cancer, and it clearly improves prognosis [1]. Multiparametric molecular assays are increasingly used to estimate prognosis and guide treatment decisions of patients with primary ER+ breast cancer. These include the Oncotype DX (OncotypeIQ/Genomic Health, Inc., Redwood City, CA, USA) Recurrence Score (RS) [2], Prosigna PAM50 (NanoString Technologies, Seattle, WA, USA) [3], Breast Cancer Index (BCI) [4], EndoPredict (Myriad Genetics, Zurich, Switzerland) [5] and IHC4 [6]. All of them have been evaluated in the TransATAC series of samples that were established from patients with ER+ primary breast cancer randomised to treatment with 5 years of anastrozole or tamoxifen in the ATAC (Arimidex, Tamoxifen, Alone or in Combination) trial [7]. It has become clear that, following surgery, the risk of recurrence in ER+ primary breast cancer is not constant, which is underlined by molecular differences. In TransATAC we have previously shown that the oestrogen module of RS was prognostic within 5 years of surgery (during endocrine therapy), however it became non-informative for recurrences beyond 5 years, thus weakening the overall prognostic value of RS [8]. In the same data set, patients with high ER expression by RT-PCR were twice as likely to have a relapse 5–10 years after surgery than within the first 5 years. Bianchini et al. reported risk stratification by integrating the mitotic kinase score (MKS) and an oestrogen receptor-related score (ERS), both based on genes constituting the proliferation and oestrogen modules of RS. Women with high MKS and ERS tumours were at greater risk of late recurrence [9]. More recently, improved risk estimation beyond 5 years by RS was reported when integrated with dichotomised ER expression assessed by RT-PCR [10].

Extending endocrine therapy beyond 5 years has been shown to reduce late-recurrence rate [11, 12], however those most likely to benefit from such therapy need to be identified. Although some of the widely used prognostic assays for ER+ patients have been shown to be prognostic for risk beyond 5 years [13–16], none of them have been optimised to quantify residual risk after 5 years free from recurrence, and their ability to predict late relapse varies substantially [17]. The different time-dependent performance of multiparametric molecular signatures indicates that molecular features of ER+ breast cancers may be identified to improve prediction of residual risk in order to spare those patients with significantly low risk of late recurrence from extended endocrine therapy.

We therefore hypothesised that prognostic signatures optimised specifically for the early (0–5 years) and late (5–10 years) follow-up periods, respectively, would be more prognostic than a single signature optimised for the whole 10-year follow-up period. To test this hypothesis, we developed time-dependent prognostic signatures in patient samples from the TransATAC series for early, late and 10-year follow-up periods. The prognostic performance was tested in an independent sample set and against commercial signatures already assessed in TransATAC. Our primary aim was to compare the prognostic value of the newly developed signature(s) added to Clinical Treatment Score (CTS) [6] with that of PAM50 risk of recurrence (ROR) based on subtype and proliferation added to CTS.

## Methods

### Patient cohorts

Our initial analysis drew from four published breast cancer cohorts (GSE6532, GSE9195, GSE17705, GSE26971) analysed on either of the Affymetrix Human Genome HG-U133A (GPL96) and HG-U133 Plus 2.0 (GPL570) microarray platforms (Affymetrix, Santa Clara, CA, USA). The two platforms shared 22,277 probes to which we restricted our analyses. This cohort had 747 unique patient samples that matched our selection criteria: ER+, HER2−, treated with 5 years of endocrine therapy, chemotherapy-naive, with information on either distant metastasis-free survival (DMFS) or relapse-free survival (RFS) available with a long follow-up. Details of the inclusion criteria are listed in Additional file 1: Methods, and a full list of samples included in the analysis is shown in Additional file 2: Table S1.

In the TransATAC cohort, RNA was available from 948 formalin-fixed, paraffin-embedded (FFPE) tumours from the ATAC trial, previously extracted by Genomic Health Inc. (GHI) [18]. Eligibility required hormone receptor-positive/HER2− disease, without chemotherapy treatment and at least 500 ng of RNA available. One hundred eighty-three recurrence events were recorded for this cohort. This study was approved by the South-East London Research Ethics Committee, and all patients gave informed consent.

The POLAR (Predictors Of early versus LAte Recurrence in ER+ breast cancer) samples were identified from archives of Royal Marsden Hospital (RMH), London, UK, and Lund University Hospital Biobank, Lund, Sweden. Eligibility criteria were patients with ER+/HER2− early breast cancer diagnosed between January 2000 and December 2004, treated with curative intent and with a follow-up data cut-off at May 2014. Patients must have received 5 years of adjuvant endocrine therapy (unless relapse occurred within this time); (neo)adjuvant chemotherapy was permitted. A 422-sample case-control design was used; control subjects were randomly selected according to matching criteria from among the remaining cohort of patients who did not relapse during follow-up. The total number of patients drawn upon was 1449. The following four matching criteria were used in this study: (1) age at diagnosis (< 50 or > 50 years), (2) Nottingham Prognostic Index (NPI) category (< 3.4, 3.4–5.4, > 5.4), (3) type of adjuvant endocrine therapy (tamoxifen only vs. any aromatase inhibitor [AI]) and (4) chemotherapy use (yes or no). Two-hundred forty-seven recurrence events were recorded. The POLAR study was approved by the RMH Research Ethics Committee (CCR 4122) and the ethics committee of Lund University Hospital (LU 240-01).

### Study endpoints

The primary endpoint was time to any recurrence, which was defined as locoregional (ipsilateral breast, contralateral breast and regional lymph nodes) and/or distant recurrence. Secondary endpoint was time to distant recurrence, which was the time from diagnosis until metastasis from the primary tumour at distant organs, excluding contralateral disease and locoregional and ipsilateral recurrences. Death before recurrence was treated as a censoring event for both endpoints.

### Analytic procedures

In the microarray data set, 454 probes representing 454 genes (Additional file 2: Table S3) were analysed at univariate level; those significant in univariate analyses in a particular setting were entered into multivariable analyses. Further details are provided in Additional file 1: Methods.

For TransATAC, RNA was extracted by GHI for the RS study [18]. RNA (100 ng) was used with the nCounter platform (NanoString Technologies) to assay the 93 endogenous and 7 reference genes selected in the process of the microarray expression analysis in 948 TransATAC samples.

For POLAR, RNA was extracted from three 3 × 10-μm unstained sections with more than 40% tumour cellularity using the RNeasy FFPE kit (Qiagen, Hilden, Germany) according to the manufacturer’s instructions. RNA was quantified by using a NanoDrop instrument (Thermo Fisher Scientific, Wilmington, DE, USA). Between 50 and 200 ng of RNA was used to profile the expression of 27 endogenous and 5 reference genes with the NanoString nCounter.

NanoString expression data were background-corrected by subtracting the mean of the eight negative control probes, normalised with the geometric mean of five reference genes that had a correlation of Pearson’s *r* > 0.8 with all endogenous genes. The data set was then logarithmically (base 2) transformed and z-score-transformed. The *KIF20A* gene was detected in < 10% of samples in the TransATAC cohort and was removed from the data set. CTS, which carries information on tumour size, nodal status, grade, age and type of endocrine therapy, was calculated as published previously [6].

We trained separate early, late and 10-year signatures by performing elastic net analysis in the TransATAC training cohort. Our objective was to test if the early and late signatures had statistically significantly more prognostic power than the 10-year signature. If so, we would test the validity of the early and late signatures in the non-chemotherapy-treated subpopulation of POLAR and also test their performance in the chemotherapy-treated POLAR cohort. If the early and late signatures were not statistically significantly more prognostic than the overall signature, we would test the validity of the overall signature in the chemotherapy-naive POLAR group and explore its performance in the chemotherapy-treated POLAR group.

Statistical analyses of the cohort with microarray data were carried out at the Institute of Cancer Research (ICR) using R version 3.03 software (R Foundation for Statistical Computing, Vienna, Austria). Statistical analyses using the TransATAC cohort were performed at Queen Mary University of London with STATA version 13.1 (StataCorp, College Station, TX, USA) and R version 3.0.3 software. Statistical work on POLAR was carried out at RMH using the Statistical Analysis Plan version 2.0 and Prism 6.0c (GraphPad Software, La Jolla, CA, USA) software. Before data analysis took place, the statistical analysis plan for the TransATAC study was approved by the Long-term Anastrozole vs Tamoxifen Treatment Effects committee and that for the POLAR study was approved by the RMH Committee for Clinical Research, and these plans are described in Additional file 1: Methods. All statistical tests were two-sided.

## Results

We performed the following steps in our study. We used publicly available microarray data to generate lists of prognostic genes to be analysed in the TransATAC cohort. We developed early, late and 10-year prognostic signatures in a training data set (two-thirds of TransATAC) while setting aside a test set (one-third of TransATAC) so that the performance of the newly trained signatures could be evaluated. This internal validation included comparison with commercial signatures of BCI, Oncotype DX RS, PAM50 ROR and IHC4. Finally, we conducted an external validation in the POLAR case-control sample set.

### Candidate gene selection and microarray expression data analysis

**Fig. 1:**
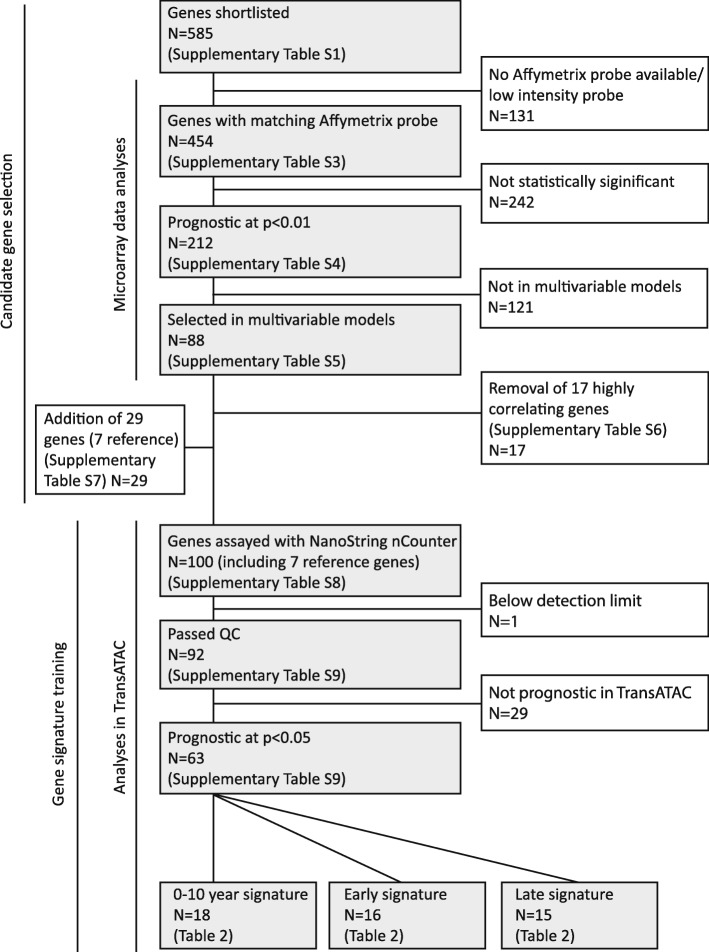
Flowchart of gene signature derivation in the microarray and TransATAC cohorts. *QC* Quality control, *ATAC* Arimidex, Tamoxifen, Alone or in Combination

In order to derive time-dependent prognostic signatures, we shortlisted 585 candidate genes representing proliferation, oestrogen signalling, immune infiltration and immune signalling. These genes were tested for prognostic significance in publicly available gene expression sets of ER+ endocrine therapy-treated breast cancer. A flowchart illustrating the approach is shown in Fig. 1. Sixty-seven genes of interest that are part of the PAM50, Oncotype DX RS, EndoPredict and BCI profilers were also included. Additional genes likely to be related to benefit from endocrine therapy were identified from 81 patients by reanalysing our previously published neoadjuvant endocrine therapy-treated set of samples [19] (https://www.synapse.org/#!Synapse:syn16243). From this dataset, we identified 164 candidate genes by examining correlation of individual gene expression from untreated biopsies with change in the following after 2 weeks of AI treatment: (1) Ki-67, (2) proliferation-associated gene cluster, (3) oestrogen-associated gene cluster, and (4) expression of the modified version of the Global Index of Dependence on Estrogen [20] genes. An additional 354 genes were selected on the basis of literature searches. Genes from published gene modules of the proliferation-associated gene cluster, oestrogen-associated gene cluster and inflammatory response signature [19], the tumour invasion/metastasis module (*PLAU*) [21] and IGG-14 module (immunoglobulin-gamma) [22] were also included. The complete list of candidate genes and the reason for their inclusion are detailed in Additional file 2: Table S1.

Seven hundred forty-seven samples from the microarray expression dataset were compiled from four publicly available breast cancer cohorts to investigate the relationship between genes and outcome (Additional file 2: Table S2) [5, 23–25]. Expression data were available for 454 genes (Additional file 2: Table S3). We performed univariate Cox proportional hazards regression analyses for early, late and 10-year follow-up periods using RFS and DMFS as endpoints, respectively (six analyses), that identified 212 genes that were significant at *p* < 0.01 in any of the analyses (Additional file 2: Table S4). Genes significantly prognostic in a particular time period were taken forward for multivariable analyses performed by Cox proportional hazards regression with DMFS and RFS as endpoints, respectively, in the early, late and 10-year follow-up settings (six analyses). This resulted in 88 genes being selected in the models (Additional file 2: Table S5), of which 17 genes were removed owing to high correlation of expression with other candidates already selected (Additional file 2: Table S6). An additional 29 genes were added that included candidates without probes available in the microarray expression data analyses, some recently emerging candidates and also seven reference genes (Additional file 2: Table S7).

### Expression profiling and signature building in TransATAC

**Fig. 2:**
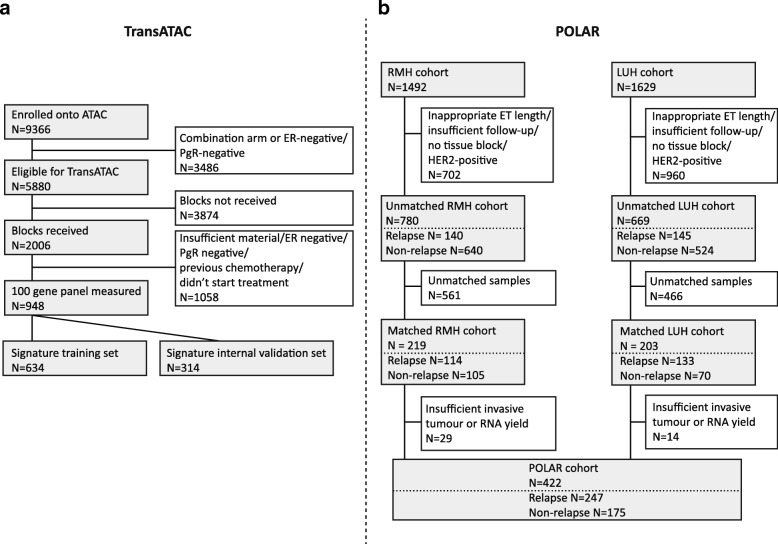
Consolidated Standards of Reporting Trials (CONSORT) diagram of the availability of samples for analysis from (**a**) the ATAC trial and (**b**) the POLAR collection of samples. *POLAR* Molecular Predictors Of early versus LAte Recurrence in ER-positive breast cancer, *ATAC* Arimidex, Tamoxifen, Alone or in Combination, *ER* Oestrogen receptor, *PgR* Progesterone receptor, *RMH* Royal Marsden Hospital, *LUH* Lund University Hospital, *ET* Endocrine therapy, *HER2* Human epidermal growth factor receptor 2

Sample availability in TransATAC is shown in Fig. 2a. Expression data for the 100 selected genes (including housekeeping genes) (Additional file 2: Table S9) were obtained for 948 patient samples in TransATAC using the NanoString nCounter. We assessed the prognostic value of these molecular variables in TransATAC for early, late and 10-year time periods for RFS. Sixty-three genes were statistically significant in at least one of the time windows assessed (Additional file 2: Table S7, Additional file 3: Figure S1). We found different prognostic properties between early and late periods for 20 genes. Six genes were prognostic early but not in the late period (*CD79*, *IL6ST*, *LRRC48*, *MPZL1*, *PGR* and *PIGV*), and 14 genes were not significantly prognostic early but gained prognostic significance in the late setting (*ANP32E*, *ANXA1*, *CTSL2*, *EPB41L2*, *ESR1*, *FOXA1*, *ICOS*, *IL17RB*, *MMP9*, *MYCBP2*, *NR2F1*, *PDZK1*, *SLAMF8* and *TCF7L2*).

The TransATAC cohort was then randomly split into two-thirds (*n* = 634) training and one-third (*n* = 314) validation sets while ensuring that the recurrence rate was similar in the two subgroups. Demographics for the training, validation and overall cohorts are presented in Table 1. We aimed to select prognostic variables independent of clinicopathological features that are commonly used for prognosis. To achieve this, on top of the 63 statistically significant genes in univariate analyses, CTS was also entered into multivariable selections for early, late and 10-year time-periods, respectively. Elastic net penalised Cox regression with leave-one-out cross-validation was used for feature selection in the TransATAC training set. CTS was selected in all three signatures in addition to 18 genes in the 10-year, 16 genes in the early, and 15 genes in the late follow-up analyses. The variables and their coefficients derived from the elastic net models are listed in Table 2. CTS had the highest coefficient in each of the time periods.

**Table 1:** Demographics of TransATAC and POLAR cohorts

	TransATAC			POLAR								
				POLAR RMH			POLAR LUH			Total POLAR (RMH + LUH)		
Patient group	Training	Validation	Total	Cases	Controls	Total	Cases	Controls	Total	Cases	Controls	Total
Number of patients	634	314	948	114	105	219	133	70	203	247	175	422
Age at diagnosis, years^a^												
Mean, years	64	65	65	56	58	57	62	60	61	59	58	59
Median, years	64	64	64	54	58	56	61	58	61	58	58	58
Range, years	48–89	47–86	47–89	29–93	28–88	28–93	35–100	35–87	35–100	29–100	28–88	28–100
Tumour size												
< 2 cm	427 (67%)	207 (66%)	634 (67%)	40 (35%)	43 (41%)	83 (38%)	40 (30%)	27 (39%)	67 (33%)	80 (32%)	70 (40%)	150 (36%)
2–5 cm	194 (31%)	101 (32%)	295 (31%)	63 (55%)	54 (51%)	117 (53%)	89 (67%)	40 (57%)	129 (64%)	152 (62%)	94 (54%)	246 (58%)
> 5 cm	13 (2%)	6 (2%)	19 (2%)	11 (10%)	8 (8%)	19 (9%)	4 (3%)	3 (4%)	7 (3%)	15 (6%)	11 (6%)	26 (6%)
Grade												
1	177 (28%)	88 (28%)	265 (28%)	12 (11%)	14 (13%)	26 (12%)	9 (7%)	11 (16%)	20 (10%)	21 (9%)	25 (14%)	46 (11%)
2	368 (58%)	169 (54%)	537 (57%)	48 (42%)	52 (50%)	100 (46%)	72 (54%)	35 (50%)	107 (53%)	120 (49%)	87 (50%)	207 (49%)
3	89 (14%)	57 (18%)	146 (15%)	54 (47%)	39 (37%)	93 (42%)	52 (39%)	24 (34%)	76 (37%)	106 (43%)	63 (36%)	169 (40%)
Histological subtype												
IDC	492 (78%)	230 (73%)	722 (76%)	80 (70%)	75 (71%)	155 (71%)	104 (78%)	55 (79%)	159 (78%)	184 (74%)	130 (74%)	314 (74%)
ILC	86 (14%)	60 (19%)	146 (15%)	22 (19%)	18 (17%)	40 (18%)	27 (20%)	15 (21%)	42 (21%)	49 (20%)	33 (19%)	82 (19%)
Other	56 (9%)	24 (8%)	80 (8%)	12 (11%)	12 (11%)	24 (11%)	2 (2%)	0 (0%)	2 (1%)	14 (6%)	12 (7%)	26 (6%)
Nodal status												
Node negative	441 (70%)	224 (71%)	665 (70%)	60 (53%)	56 (53%)	116 (53%)	42 (32%)	29 (41%)	71 (35%)	102 (41%)	85 (49%)	187 (44%)
1–3 positive nodes	136 (22%)	63 (20%)	199 (21%)	25 (22%)	34 (32%)	59 (27%)	54 (41%)	31 (44%)	85 (42%)	79 (32%)	65 (37%)	144 (34%)
4 or more nodes	57 (9%)	27 (9%)	84 (9%)	29 (25%)	15 (14%)	44 (20%)	37 (28%)	10 (14%)	47 (23%)	66 (27%)	25 (14%)	91 (22%)
PgR												
Negative	114 (18%)	46 (15%)	160 (17%)	5 (4%)	7 (7%)	12 (5%)	25 (19%)	9 (13%)	34 (17%)	30 (%)	16 (%)	46 (11%)
Positive	513 (81%)	268 (85%)	781 (82%)	20 (18%)	23 (22%)	43 (20%)	102 (77%)	56 (80%)	158 (78%)	122 (%)	79 (%)	201 (48%)
Unknown	7 (1%)	–	7 (1%)	89 (78%)	75 (71%)	164 (75%)	6 (5%)	5 (7%)	11 (5%)	95 (%)	80 (%)	175 (41%)
NPI category^a^												
≤ 3.4	298 (47%)	140 (45%)	438 (46%)	25 (22%)	26 (25%)	51 (23%)	15 (11%)	13 (19%)	28 (14%)	40 (16%)	39 (22%)	79 (19%)
3.4–5.4	281 (44%)	147 (47%)	428 (45%)	49 (43%)	50 (48%)	99 (45%)	77 (58%)	42 (60%)	119 (59%)	126 (51%)	92 (53%)	218 (52%)
> 5.4	55 (9%)	27 (9%)	82 (9%)	40 (35%)	29 (28%)	69 (32%)	41 (31%)	15 (21%)	56 (28%)	81 (33%)	44 (25%)	125 (30%)
Endocrine therapy^a^												
Tamoxifen only	301 (47%)	163 (52%)	464 (49%)	80 (70%)	72 (69%)	152 (69%)	96 (72%)	50 (71%)	146 (72%)	176 (71%)	122 (70%)	298 (71%)
AI	333 (53%)	151 (48%)	484 (51%)	34 (30%)	33 (31%)	67 (31%)	37 (28%)	20 (29%)	57 (28%)	71 (29%)	53 (30%)	124 (29%)
Chemotherapy^a^												
No	634 (100%)	314 (100%)	948 (100%)	54 (47%)	49 (47%)	103 (47%)	94 (71%)	55 (79%)	149 (73%)	148 (60%)	104 (59%)	252 (60%)
Yes	0	0	0	60 (53%)	56 (53%)	116 (53%)	39 (29%)	15 (21%)	54 (27%)	99 (40%)	71 (41%)	170 (40%)

*Abbreviations: AI* Aromatase inhibitor, *IDC* Invasive ductal carcinoma, *ILC* Invasive lobular carcinoma, *PgR* Progesterone receptor, *NPI* Nottingham Prognostic Index, *RMH* Royal Marsden Hospital, *LUH* Lund University Hospital, *POLAR* Molecular Predictors Of early versus LAte Recurrence in ER+ breast cancer

^a^ Denotes matching criteria in POLAR

**Table 2:** Variables and corresponding beta-coefficients of the time-dependent 10-year, early and late signatures

Variable	10-Year signature	Early signature	Late signature
ALDH1A1			−0.194
ANP32E	0.143	0.010	0.083
CRABP2	0.084	0.207	
CXCL12		−0.183	
CXCR4	0.142		0.056
EGFR			−0.030
ELF5	−0.046	−0.001	
FGF2	−0.178		−0.232
IGF1	−0.029	−0.017	
IGJ	−0.086	−0.037	−0.030
IL6ST		−0.044	
LINC00341	− 0.463	− 0.362	− 0.392
LRRC48		− 0.104	
MMP9	0.043		0.064
MPZL1	0.276	0.066	0.043
NUSAP1	0.088	0.065	
PBX1	0.159		0.375
PDZK1	−0.011		−0.063
PGR		− 0.073	
PRC1		0.019	
RGL1	−0.429	− 0.166	− 0.161
RRM2	0.077	0.124	
SFRP1	−0.017		−0.278
STC2	−0.087	−0.068	
TNF	−0.029		−0.026
ZEB2			−0.138
CTS	0.514	0.409	0.516

Positive coefficients are associated with higher recurrence risk; negative coefficients are associated with lower recurrence risk. Beta-coefficients were normalised by dividing them by the SD of the respective variables in the training population

### Comparison of time period-optimised prognostic signatures in TransATAC validation set

TransATAC was used to validate and compare the prognostic information of the three time period-dependent signatures (Table 3). In the 0–10-year follow-up period, all three newly derived signatures were significantly prognostic, with the late signature being significantly less informative than the 10-year signature (10-year signature likelihood ratio chi-square test [LRχ^2^] = 28.0; early signature LRχ^2^ = 33.4; late signature LRχ^2^ = 18.1). In the 0–5-year period, the 10-year signature and early signature were equally prognostic and significantly more than the late signature (LRχ^2^ for 10-year signature = 14.1; LRχ^2^ for early signature = 14.9; LRχ^2^ for late signature = 8.9). In the late setting, the early signature was the most prognostic, followed by the 10-year and late signatures (LRχ^2^ for 10-year signature = 13.9; LRχ^2^ for early signature = 18.6; LRχ^2^ for late signature = 9.3). CTS was strongly prognostic in all three time periods (CTS 0–10-year LRχ^2^ = 48.7; CTS 0–5-year LRχ^2^ = 29; CTS 5–10-year LRχ^2^ = 19.8).

**Table 3:** Statistical analysis of TransATAC validation cohort

Score	No. of patients (relapses)	Univariate comparisons					Multivariable comparisons				
							CTS + signature vs CTS				CTS + signature
		LRχ^2^	*p* Value	HR (95% CI)	P diff	C-index (SE)	ΔLRχ^2^	*p* Value	HR (95% CI)	P diff	C-index (SE)
0–10 years											
CTS	314 (59)	48.7	< 0.001	2.16 (1.79–2.62)	–	0.674 (0.018)	–	–	–	–	–
10-Year signature		28	< 0.001	1.98 (1.54–2.55)	Reference	0.671 (0.026)	7.9	0.005	1.49 (1.13–1.96)	Reference	0.709 (0.021)
Early signature		33.4	< 0.001	2.06 (1.62–2.61)	0.334	0.678 (0.024)	10.3	0.001	1.55 (1.19–2.02)	0.48	0.711 (0.020)
Late signature		18.1	< 0.001	1.72 (1.34–2.20)	0.000	0.642 0(.029)	4.3	0.037	1.33 (1.02–1.74)	0.004	0.700 (0.022)
0–5 years											
CTS	314 (26)	29	< 0.001	2.04 (1.53–2.74)	–	0.679 (0.023)	–	–	–	–	–
10-Year signature		14.1	< 0.001	2.05 (1.41–2.98)	0.833	0.678 (0.037)	3.2	0.073	1.46 (0.97–2.19)	0.77	0.712 (0.029)
Early signature		14.9	< 0.001	2.00 (1.42–2.81)	Reference	0.672 (0.035)	2.8	0.096	1.40 (0.95–2.06)	Reference	0.705 (0.028)
Late signature		8.9	0.003	1.77 (1.22–2.57)	0.138	0.648 (0.042)	1.7	0.19	1.31 (0.87–1.97)	0.65	0.705 (0.031)
5–10 years											
CTS	270 (33)	19.8	< 0.001	1.84 (1.33–2.54)	–	0.657 (0.026)	–	–	–	–	–
10-Year signature		13.9	< 0.001	1.93 (1.37–2.72)	0.027	0.663 (0.036)	4.8	0.028	1.53 (1.05–2.22)	0.14	0.696 (0.030)
Early signature		18.6	< 0.001	2.11 (1.52–2.94)	0.091	0.681 (0.032)	8	0.005	1.70 (1.19–2.43)	0.14	0.708 (0.028)
Late signature		9.3	0.002	1.68 (1.21–2.34)	Reference	0.636 (0.038)	2.7	0.099	1.36 (0.95–1.94)	Reference	0.686 (0.031)

*CTS* Clinical Treatment Score, *LR* Likelihood ratio

Both univariate and multivariable analyses are presented for years 0–10, years 0–5, and years 5–10 separately. Likelihood ratio test based on Cox proportional hazards models for univariate and multivariable analyses. Differences in likelihood ratio values (ΔLRχ^2^) were used. CTS was used as a covariate in the multivariable regressions. For each score, HRs per SD change are presented

For the 0–10-year period, all three signatures added statistically significant prognostic information beyond that of the CTS (ΔLRχ^2^ for 10-year signature = 7.9; ΔLRχ^2^ for early signature = 10.3; ΔLRχ^2^ for late signature = 4.3). In the 0–5-year period none of the signatures added significant prognostic information to CTS. However, in the 5–10-year period, the 10-year and early signatures added statistically significant prognostic information to CTS (10-year signature ΔLRχ^2^ = 4.8; early signature ΔLRχ^2^ = 8.0; late signature ΔLRχ^2^ = 2.7).

Given that the early and the late signatures were not statistically significantly more prognostic than the 10-year signature in the respective periods they were optimised for, we rejected our primary hypothesis that signatures optimised separately for the early and the late follow-up periods, respectively, are more prognostic than a 10-year signature, but we proceeded to assess the validity of the 18-gene, 10-year signature in an independent cohort and to compare its performance with that of commercial signatures.

### Signature test of 10-year validity in POLAR cohort

A matched case-control set of samples was compiled from RMH and Lund University Hospital archives (POLAR) to validate the 10-year signature (Fig. 2b, Table 1). Our aims were to test the validity the 10-year signature in an endocrine therapy-only cohort similar to the training set and also to explore if the prognostic property (if any) extends to a higher-risk, chemotherapy-treated population. The latter cohort was of interest in the 5–10-year period because of the potential for its use in selecting patients for extended adjuvant endocrine therapy.

Despite having matched cases and controls on NPI category, the CTS was still higher in cases than in control subjects: 201.9 ± 98 (SD) vs. 170.8 ± 87.6 (*p* = 0.0009), respectively. In a univariate analysis, CTS had an OR of 1.004 (95% CI, 1.001–1.006) for a one-unit increase. We assessed a multivariable model with CTS with and the 10-year signature, and both were found to be statistically significant: 10-year signature OR = 1.851 (95% CI, 1.194–2.868), *p* = 0.006; CTS OR = 1.003 (1.001–1.005), *p* = 0.012.

We also assessed whether the 10-year signature added significant prognostic information above CTS alone using LR tests (Table 4, Additional file 4: Table S10). In the overall POLAR cohort (*n* = 422), CTS was prognostic across 10 years and in the early follow-up period (CTS 0–10-year period LRχ^2^ = 11.23; 0–5-year period LRχ^2^ = 22.09), but not in the 5–10-year period. The 10-year signature was prognostic in all three follow-up periods and contributed to CTS with significant prognostic information in the 10-year and early periods (0–10-year period ΔLRχ^2^, CTS + 10-year signature vs. CTS = 7.74; 0–5-year period ΔLRχ^2^, CTS + 10-year signature vs. CTS = 7.59), but not in the 5–10-year period. Both CTS and the 10-year signature were marginally more informative across the 10 years in the chemotherapy-treated POLAR cohort than in the endocrine therapy-only population, despite the latter having more patients and events (patients, *n* = 170 vs. *n* = 252; events, 99 vs. 148). Additionally, the 10-year signature added significantly more prognostic information to CTS in the chemotherapy-treated group (ΔLRχ^2^: CTS + 10-year signature vs. CTS = 6.71) than among those receiving endocrine therapy only (ΔLRχ^2^, CTS + 10-year signature vs. CTS = 2.47).

**Table 4:** Statistical analysis of three groups of POLAR validation set for 0–10 years of follow-up

			All POLAR patients	Chemotherapy-treated	Chemotherapy-naive
0–10 Years					
No. of patients (relapses)			*n* = 422 (247)	*n* = 170 (99)	*n* = 252 (148)
Univariate	CTS	LRχ^2^	11.23	7.75	6.1
		P	< 0.001	0.005	0.014
		C-index (SE)	0.594 (0.028)	0.623 (0.044)	0.590 (0.036)
	10-Year signature	LRχ^2^	12.44	7.73	5.39
		P	< 0.004	0.005	0.020
		C-index (SE)	0.593 (0.028)	0.619 (0.044)	0.578 (0.037)
Multivariable comparisons	CTS + 10-year signature vs CTS	ΔLRχ^2^	7.74	6.71	2.47
		P	0.005	0.001	0.116
	CTS + 10-year signature	C-index (SE)	0.617 (0.028)	0.669 (0.042)	0.598 (0.036)

*Abbreviations: POLAR* Molecular Predictors Of early versus LAte Recurrence in ER-positive breast cancer, *CTS* Clinical Treatment Score, *LR* Likelihood ratio, *SE* standard error

Both univariate and multivariable analyses are presented for years 0–10, years 0–5, and years 5–10 separately. Likelihood ratio test based on Cox proportional hazards models for univariate and multivariable analyses. Differences in likelihood ratio values (ΔLRχ^2^) were used. CTS was used as a covariate in the multivariable regressions

**Fig. 3:**
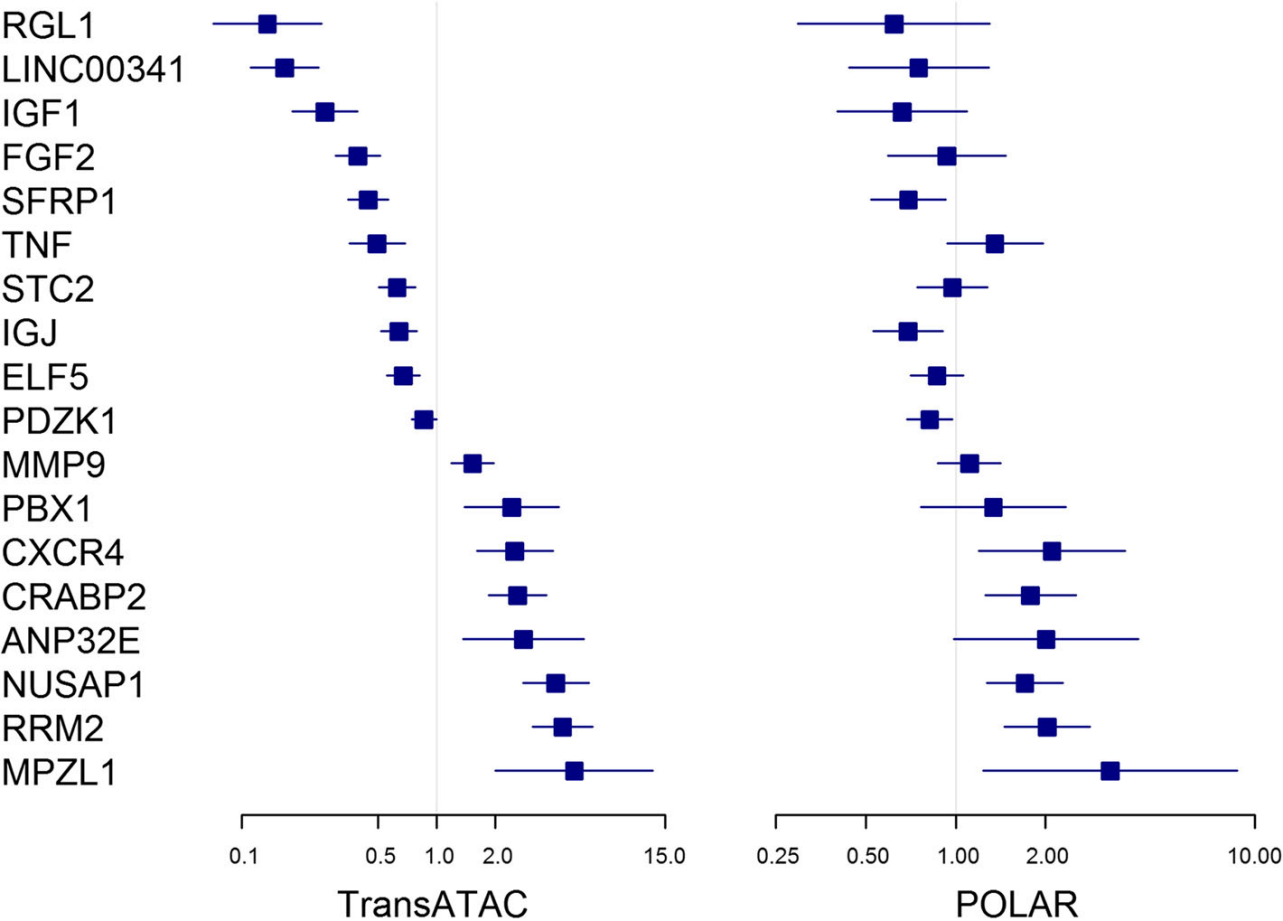
HRs and ORs for the 10-year signature genes in TransATAC and POLAR, respectively. *POLAR* Molecular Predictors Of early versus LAte Recurrence in ER-positive breast cancer, *ATAC* Arimidex, Tamoxifen, Alone or in Combination

Prognostic properties of the 18 individual genes constituting the 10-year signature were assessed in POLAR and compared with data obtained in TransATAC. In POLAR, only 8 of the 18 genes were significantly prognostic at the univariate level (Fig. 3), but all genes except tumour necrosis factor-alpha (TNF) showed the same prognostic direction both in TransATAC and in POLAR.

### Comparison of the 10-year signature with CTS, RS, PAM50 ROR, BCI and IHC4 in TransATAC

We have previously published data on the prognostic performance of CTS, RS, PAM50 ROR, BCI and IHC4 in TransATAC [6, 15, 18, 26]; data for all scores were available for 271 patients in the validation cohort. We assessed their prognostic information for 10 years after surgery using any recurrence and distant recurrence as endpoints, respectively, and compared them with the newly developed 10-year signature (Table 5). For both any and distant recurrence, the BCI provided the most added information beyond the CTS in this set (any recurrence, CTS LRχ^2^ = 37.4; BCI ΔLRχ^2^ = 9.5; distant recurrence, CTS LRχ^2^ = 46.7; BCI ΔLRχ^2^ = 14.5, respectively). The novel 10-year signature performed similarly to the other three scores in this respect.

**Table 5:** Statistical analysis for all and distant recurrences in the TransATAC validation cohort

Score	Univariate				Multivariable comparisons			
					CTS + signature vs CTS			CTS + signature
	LRχ^2^	*p* Value	HR (95% CI)	C-index (SE)	∆LRχ^2^	*p* Value	HR (95% CI)	C-index (SE)
All recurrences (*n* = 271, AR = 55)								
CTS	37.4	< 0.001	1.94 (1.57–2.40)	0.664 (0.020)	–	–	–	–
10-Year signature	20.7	< 0.001	1.85 (1.42–2.41)	0.657 (0.029)	5.7	0.017	1.42 (1.07–1.89)	0.695 (0.023)
BCI	25.0	< 0.001	2.07 (1.54–2.77)	0.679 (0.029)	9.5	0.002	1.62 (1.19–2.21)	0.711 (0.024)
RS	11.1	< 0.001	1.52 (1.21–1.91)	0.607 (0.027)	5.8	0.016	1.35 (1.07–1.71)	0.683 (0.021)
ROR	18.3	< 0.001	1.77 (1.36–2.31)	0.650 (0.030)	6.0	0.014	1.42 (1.07–1.87)	0.700 (0.024)
IHC4	14.4	< 0.001	1.63 (1.28–2.10)	0.629 (0.029)	7.5	0.006	1.46 (1.12–1.91)	0.696 (0.023)
Distant recurrences (*n* = 271, DR = 41)								
CTS	46.7	< 0.001	2.25 (1.79–2.82)	0.689 (0.019)	–	–	–	–
10-Year signature	26.4	< 0.001	2.24 (1.64–3.06)	0.694 (0.029)	8.5	0.004	1.65 (1.18–2.30)	0.733 (0.023)
BCI	34.0	< 0.001	2.71 (1.91–3.84)	0.726 (0.028)	14.5	< 0.001	2.03 (1.40–2.95)	0.754 (0.023)
RS	10.7	< 0.001	1.58 (1.23–2.03)	0.616 (0.029)	5.1	0.024	1.38 (1.06–1.79)	0.707 (0.020)
ROR	21.3	< 0.001	2.05 (1.50–2.79)	0.680 (0.031)	7.5	0.006	1.58 (1.14–2.21)	0.736 (0.024)
IHC4	17.9	< 0.001	1.87 (1.41–2.49)	0.658 (0.031)	9.8	0.002	1.68 (1.22–2.31)	0.731 (0.023)

*Abbreviations: AR* All recurrences, *DR* Distant recurrences, *CTS* Clinical Treatment Score, *BCI* Breast Cancer Index, *RS* Recurrence score, *ROR* Risk of recurrence, *LR* Likelihood ratio

Both univariate and multivariable analyses are presented. For each score, HRs per SD change are presented. Likelihood ratio test based on Cox proportional hazards models for univariate and multivariable analyses. Differences in likelihood ratio values (ΔLRχ^2^) were used. CTS was used as a covariate in the multivariable regressions

## Discussion

We developed novel time-specific prognostic signatures for early, late and 10-year follow-up periods for ER+/HER2− patients treated with endocrine therapy alone to allow us to test the hypothesis that sequentially applying early and late signatures could be more prognostic for risk of relapse than a single newly developed 10-year signature. This hypothesis was based largely on our observation that the performance of some components in many of the commercially available signatures varied between these time periods. For example, we found that ESR1 and the oestrogen module overall in the RS was less prognostic in years 5–10 than in years 0–5 [8]. Analogous findings were reported by Bianchini et al. [9]. Very recently, the Early Breast Cancer Trialists’ Collaborative Group (EBCTCG) published data on clinicopathological and limited immunohistochemical data on over 60,000 women who were treated with 5 years of endocrine therapy [27]. Although progesterone receptor showed strong prognostic performance in years 0–5, it showed no significant relationship with prognosis thereafter. These data on markers associated with hormone responsiveness support the contention, but by no means prove, that cessation of endocrine treatment at 5 years may lead to increased recurrence risk in more hormonally responsive tumours. We therefore included in our assessment genes that we and others have found to be associated with the anti-proliferative response of primary ER+ breast cancer to oestrogen deprivation. Our work involved a discovery set of 747 samples; training and test sets of 634 and 314 TransATAC samples, respectively; and independent case-control series from 1449 eligible samples. As such, this was one of the largest original gene expression analyses undertaken for evaluating prognosis in ER+ breast cancer.

Of the 92 genes selected from microarray data and assessed in univariate analyses in TransATAC, we found 63 to be significantly prognostic (*p* < 0.05) in any of the three time periods, which is considerably more than expected by chance after allowing for multiple testing errors. For most genes, the same prognostic pattern was observed for early and late periods, however we observed some possibly different prognostic properties for 20 genes. Notably, consistent with the above arguments, higher levels of ESR1 and its pioneer factor FOXA1 showed a shift at 5 years to be associated with worse prognosis beyond 5 years, but surprisingly over the 10-year period, the two genes were associated with poor prognosis. The complementary role whereby upon stimulus ER binding to chromatin is dependent on the presence of FOXA1 is well established [28]. In our dataset, FOXA1 and ESR1 correlated highly (Pearson’s *R* = 0.65); the possibility that increased expression of one or both may put patients at increased risk of late relapse merits further investigation, particularly with regard to whether the genes also identify patients who benefit from extended adjuvant therapy.

The optimised time-dependent signatures derived in the TransATAC training set were rather similar to one another in makeup. All genes in the 10-year signature featured in either (or both) of the early and late signatures with their coefficients being in the same direction. The early and late signatures had five and three variables, respectively, not present in the 10-year signature, suggesting that the early and late signatures may not have captured time-specific features or that such time-specific features that exist exert a minor modulatory influence on the overall prognosis over 10 years. It is notable that CTS was consistently the most prognostic variable in the three time-dependent models and that its contribution was similar in both early and late recurrence. This is consistent with the data of the EBCTCG that classical clinicopathological features retain their strong prognostic influence beyond 5 years [27].

Given that the 10-year signature captured prognostic features of both early and late events, it is perhaps not surprising that no improvement was seen in the use of early and late signatures compared with the overall 10-year signature that led to the rejection of our hypothesis. Also, it should be noted that splitting of the 0–10-year time period into 0–5- and 5–10-year periods markedly reduces the power to detect prognostic contributions. At least a contributory factor for the lack of improvement may be the dominance of proliferation-related genes in our and other signatures. As shown in our earlier analysis of the RS, each of the individual proliferation genes and the integrated module are equally prognostic before and after 5 years [8]. Notably, this is also supported by the observation by the EBCTCG that Ki-67 was equally prognostic before and after 5 years in their overview analysis of late recurrence [27].

The 10-year signature was nonetheless validated in the POLAR sample set and provided significant prognostic information in both chemotherapy-naive and chemotherapy-treated cohorts. Moreover, it added independent prognostic information beyond that of CTS in the POLAR cohort. Comparison of the information provided by each gene showed that 8 of the 18 genes were significantly prognostic at univariate level in POLAR (4 genes at *P* < 0.05, 2 genes at *P* < 0.01 and 3 genes at *P* < 0.001). TNF showed an opposite prognostic direction in training and validation sets, thus weakening the performance of the signature in POLAR. TNF is a versatile pro-inflammatory cytokine that has both pro- and anti-tumour activities promoting lymphocytic infiltration and activating the nuclear factor-κB, c-Jun N-terminal kinase and mitogen-activated protein kinase pathways, and it is capable of inducing apoptosis through TNF receptors 1 and 2 [29]. It may be that the inclusion of higher-risk, chemotherapy-treated patients in POLAR contributed to the difference in TNF’s prognostic pattern; further investigation is needed to explain the relationship of TNF and risk of relapse in these cohorts.

The 10-year signature was compared with established prognostic signatures in the TransATAC validation set. Importantly, the 10-year signature was developed for the endpoint of any recurrence contrary to the endpoint of distant recurrence used in the development of RS, PAM50, ROR, BCI and IHC4. In univariate assessments, BCI and the 10-year signatures were the most informative for both all and distant recurrence. When added to CTS, all signatures assessed provided similar amounts of information, with CTS + BCI being the most informative for distant recurrence. This new signature did not outperform the established signatures, even though it was based on a large and wide-ranging analysis of both established prognostic genes and novel genes with a clear rationale for inclusion. Larger studies may be needed to fully optimise novel prognostic signatures with improved prognostic information, however the data from our studies indicate that the gain is unlikely to be large. Other approaches that assess response to treatment or integrate mutational and DNA copy number profiles or by the use of circulating tumour DNA are likely to be more fruitful.

The results presented here support the mounting evidence that better risk estimation can be achieved by combining molecular profilers with clinicopathological factors. For the three time-dependent signatures derived in TransATAC, CTS was the most prognostic in all three time-dependent signatures and provided more prognostic information than RS, ROR, BCI and IHC4, respectively. Additionally, all profilers added significant prognostic information to CTS, leading to combined signatures being significantly more informative. There is emerging evidence for genetic differences affecting outcome amongst various racial groups [30]. Although this is an important question with practical consequences, the cohort presented here was > 99% Caucasian and did not provide us with the opportunity to examine within TransATAC.

Our study has strengths and limitations. An advantage was that a large discovery cohort of 634 samples was used for signature training. All tumours were ER+/HER2− from post-menopausal patients who had received 5 years of endocrine therapy without chemotherapy. This was a homogeneous group of breast cancers, which reduced confounding factors such as tumour subtype and differing treatment lengths and types. Data for the clinical prognostic tests were obtained by the same methods as set out by the tests’ developers. The same batch of RNA was used for the newly developed signatures presented here and for the clinical prognostic tests used in the comparisons, reducing intra-sample variation. The clinical data were derived from a registration standard trial with comprehensive follow-up over 10 years. Limitations include that the candidate gene selection based on microarray data and associated clinical information from multiple studies did not allow the assessment of candidates by taking multiple clinical variables into account; this may have limited the performance of derived signatures that ultimately included CTS as a variable. Also, CTS, IHC4 and the 10-year signature were derived in TransATAC; therefore, their performance in the comparisons was slightly overestimated compared with what we would see in independent cohorts. Finally, although this study was relatively large compared with others, the splitting of the data into early and late signatures decreased the statistical power for comparisons within those time periods. The approach we have taken is likely to have somewhat overfitted the 10-year signature to the TransATAC population. An alternative approach for the derivation and validation of the 10-year signature would have been to fit the signature to the whole of the TransATAC cohort and validate it in the POLAR cohort. However, the approach we took allowed the comparison of the 10-year signature with commercially available signatures in the TransATAC test set. Had the 10-year signature not at least matched these, it would not have been worth proceeding further.

## Conclusions

In summary, we found that early and late signatures are unlikely to be more informative for predicting relapse than a single signature optimised for 10 years. Larger studies may be needed to fully optimise novel gene expression signatures for prognosis in endocrine-treated ER+ patients with breast cancer, however a substantial improvement in performance is unlikely.

## Additional files

Additional file 1: Methods. Additional methods. (DOCX 21 kb)

Additional file 2: **Table S1.** List of 585 candidate genes. **Table S2.** List and identifiers for the 747-patient microarray expression data cohort. **Table S3.** List of 454 Affymetrix probes studied. **Table S4.** List of 212 genes significantly prognostic (*p* < 0.01) in any of the three time periods in the microarray data. **Table S5.** List of 88 genes by multivariable selections in any of the three time periods in the microarray data. Nodal status was used as a covariate in the regressions. **Table S6.** List of 17 genes manually removed from the multivariable list. **Table S7.** List of 29 genes added to the candidate list. **Table S8.** Details of the 100-probe NanoString code set used in TransATAC. **Table S9.** HRs, CIs and *p* values for the 92 genes assessed in TransATAC in univariate analyses. (XLSX 130 kb)

Additional file 3: **Figure S1.** Forest plot of HRs and CIs for the 92 genes assessed in TransATAC in univariate analyses. Asterisk denotes significance. (PDF 1497 kb)

Additional file 4: **Table S10.** Likelihood ratio (LR) χ^2^ and *p* values for CTS and 10-year signature in three groups of POLAR validation set for 0–5 and 5–10 years of follow-up. Both univariate and multivariable analyses are presented for years 0–10, years 0–5, and years 5–10 separately. LR test based on Cox proportional hazards models for univariate and multivariable analyses. Differences in LR values (ΔLRχ^2^) were used. CTS was used as a covariate in the multivariable regressions. *POLAR* Molecular Predictors Of early versus LAte Recurrence in ER-positive breast cancer, *CTS* Clinical Treatment Score. (DOCX 15 kb)

## Abbreviations

AI: Aromatase inhibitor
ATAC: Arimidex, Tamoxifen, Alone or in Combination
BCI: Breast Cancer Index
CTS: Clinical Treatment Score
DMFS: Distant metastasis-free survival
EBCTCG: Early Breast Cancer Trialists’ Collaborative Group
ER: Oestrogen receptor
ERS: Oestrogen receptor-related score
FFPE: Formalin-fixed, paraffin-embedded
GHI: Genomic Health Inc.
HR: Hormone receptor
IHC4: Score based on four IHC markers
LR: Likelihood ratio
LUH: Lund University Hospital
MKS: Mitotic kinase score
NPI: Nottingham Prognostic Index
POLAR: Predictors Of early versus LAte Recurrence in ER+ breast cancer
RFS: Relapse-free survival
RMH: Royal Marsden Hospital
ROR: Risk of recurrence
RS: Oncotype DX Recurrence Score
TNF: Tumour necrosis factor
