# Supplementary material for: Novel 18-gene signature for predicting relapse in ER-positive, HER2-negative breast cancer
Source: Breast Cancer Res. 2018 Sep 4;20:103. doi: 10.1186/s13058-018-1040-9 (PMC6122470; doi:10.1186/s13058-018-1040-9)
Supplement: Supplementary file 1 — Methods. Additional methods. (DOCX 21 kb) [file 13058_2018_1040_MOESM1_ESM.docx]

**Additional file 1: methods**

**Patient cohorts**

Microarray data cohort: 102 duplicate samples that were originally published in GSE6532 and subsequently reanalysed under GSE17705 were included in our cohort only once. The ER status of samples in GSE6532, GSE9195 and GSE17705 cohorts were defined in the original publications by immunohistochemistry. For GSE26971, probe intensity of 205225_at (ESR1) was used with the cut-off of 1000 also used in the original publication resulting in the removal of 7 samples from GSE26971. The HER2 status in all four cohorts was defined by using HER2 probe intensity (216836_s_at) with the cut-off of 6000. Forty-four samples were found to be HER2-positive by this criterion and subsequently removed from the cohort. In the 0-10 year follow-up period 741 samples had 168 DMFS events; the 318 samples with RFS data available had 83 relapses recorded. Nodal status was available for 696 samples.

**Analytic procedures**

For POLAR archival formalin fixed paraffin-embedded (FFPE) tissue blocks from either surgical excision specimens or core biopsies were identified. For patients who had been treated with neoadjuvant therapy, the diagnostic core was used for analysis. Oestrogen-receptor (ER), progesterone receptor (PgR) and HER2 status were determined from histopathology reports at diagnosis. For patients in whom HER2 status was unknown at the original diagnosis, HER2 staining was performed initially by immunohistochemistry (IHC) (graded from 0 to 3+) with confirmation of HER2 2+ tumours by in situ-hybridization (D-DISH). If tumours were subsequently identified as HER2-positive, they were excluded from the cohort.

**Statistical methods**

For the microarray study raw expression data of 22,277 probes was MAS5.0 normalized on a platform basis using the justMAS function in the simpleaffy R library to a mean target intensity of 600, without background correction. The two normalised datasets were then merged. Cross-cohort batch effects were corrected using the COMBAT (sva R package, Surrogate Variable Analysis) empirical Bayes method [[1](#_ENREF_1)], directly removing known batch effects. Expression probes that had a <10% intensity of all probes were removed. Seventy-five genes had no corresponding probes in the assay, 510 genes had 933 probes associated in the assay, of which for each gene the highest variance was selected. Cox Proportional Hazard was used with both continuous and median split expression to identify significantly prognostic genes. Hazard ratios and Odds ratios were derived from the standard deviation of the Cox-model regression coefficient. Analyses were performed in the early, late and 10 year time periods with distant metastasis-free survival (DMFS) and relapse-free survival (RFS) as endpoints respectively (six analyses). Statistically significant genes in univariate analyses were entered into multivariable Cox proportional hazards models.

In TransATAC the 92 genes were evaluated in the 948 sample set by continuous univariate Cox Proportional Hazard, genes significant at p<0.05 were taken forward for signature generation. For this, the 948 patients were randomly split into 2/3 training (n=634) and 1/3 validation (n=314) sets. Number of events were split similarly, nodal status and tumour size were matched between training and validation sets. Genes statistically significant in univariate analyses in either early, late or 10-year periods and clinical treatment score (CTS) were entered into multivariable selection process. Elastic net penalised Cox regression was used for feature selection with leave one out cross-validation. The minimum partial likelihood deviance was estimated for different alpha values. This was done by varying the lambda tuning parameter, that controls the overall level of shrinkage. Leave-one-out cross-validation of the partial likelihood deviance was used to estimate the best lambda. The partial likelihood deviance given alpha and lambda was obtained by getting a 1 model to all data except one observation. Then the deviance difference was between all data, and a calculation not using the observation left out. This was repeated for all data points. The lambda for each alpha was chosen was based on a `one-standard-error' rule. This selects the model with deviance one standard error away from the minimum. Alpha was set at 0.2 for all three model selections. Composite scores were built using the selected features and the beta-coefficients were determined. Beta-coefficients were normalised by dividing them by the standard deviation of the respective variables in the training population. For signature comparison C-indices and standard errors were presented [[2](#_ENREF_2)].

In POLAR the primary analyses were performed on all POLAR patients for which NanoString data was available and passed QC criteria. A control was defined as a patient who did not relapse during follow up. Controls were randomly selected according to matching criteria from the remaining cohort of patients who did not relapse during follow-up. The four matching criteria used in this study were: (i) age at diagnosis (≤50 years, >50 years), (ii) Nottingham Prognostic Index (NPI) category (<3.4; 3.4-5.4, >5.4), (iii) type of adjuvant endocrine therapy (tamoxifen only, any aromatase inhibitor), (iv) chemotherapy use (yes, no). All parametric unpaired t-tests were performed using PRISM software (Version 6.0c). Conditional logistic regression was performed using STATA to test whether the 10-year signature, CTS and individual genes were associated with risk of recurrence in a non-pairwise fashion. A multivariable conditional logistic regression analysis was performed to see whether CTS and the 10-year signature were independent variables in a forward selection manner with a 5% significance level in a non-pairwise fashion. A log likelihood test was used to test the 10-year signature and CTS score in a model provides a better fit than CTS alone with a 5% significance level. The two separate hospital cohorts constituting POLAR were also analysed separately, no significant difference in the results was found (data not shown).

**References**

1. Johnson WE, Li C, Rabinovic A: **Adjusting batch effects in microarray expression data using empirical Bayes methods**. *Biostatistics* 2007, **8**(1):118-127.

2. Gonen M, Heller G: **Concordance probability and discriminatory power in proportional hazards regression**. *Biometrika* 2005, **92**(4):965-970.
