# Supplementary figures and images for: Novel 18-gene signature for predicting relapse in ER-positive, HER2-negative breast cancer
Source: Breast Cancer Res. 2018 Sep 4;20:103. doi: 10.1186/s13058-018-1040-9 (PMC6122470; doi:10.1186/s13058-018-1040-9)

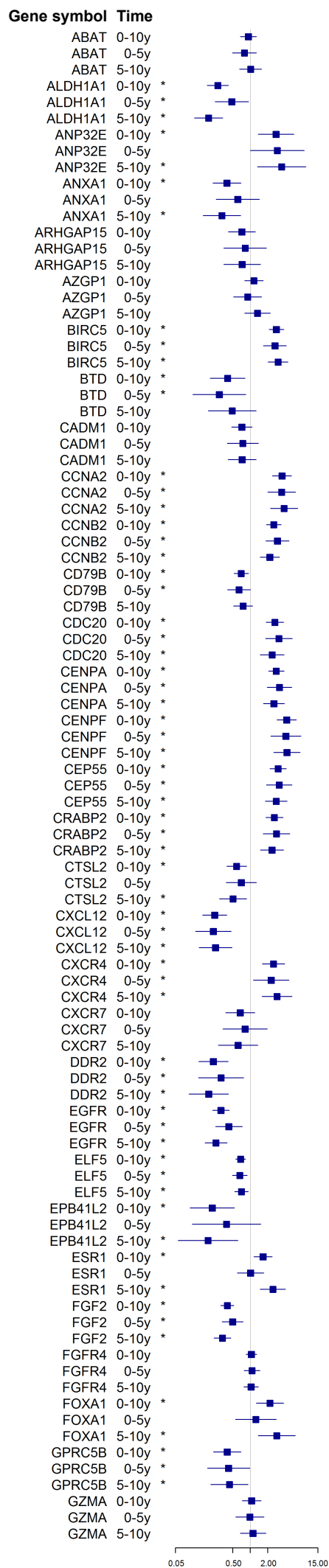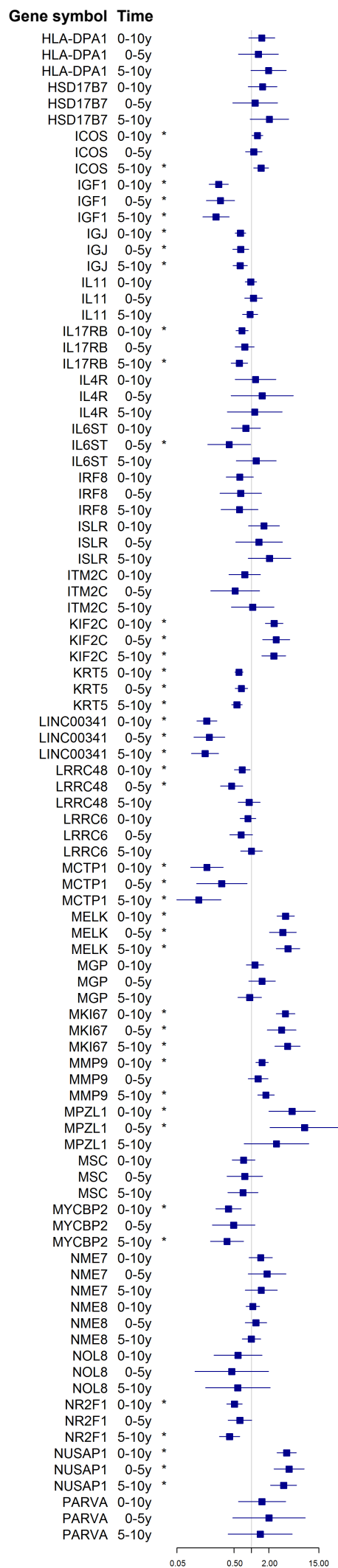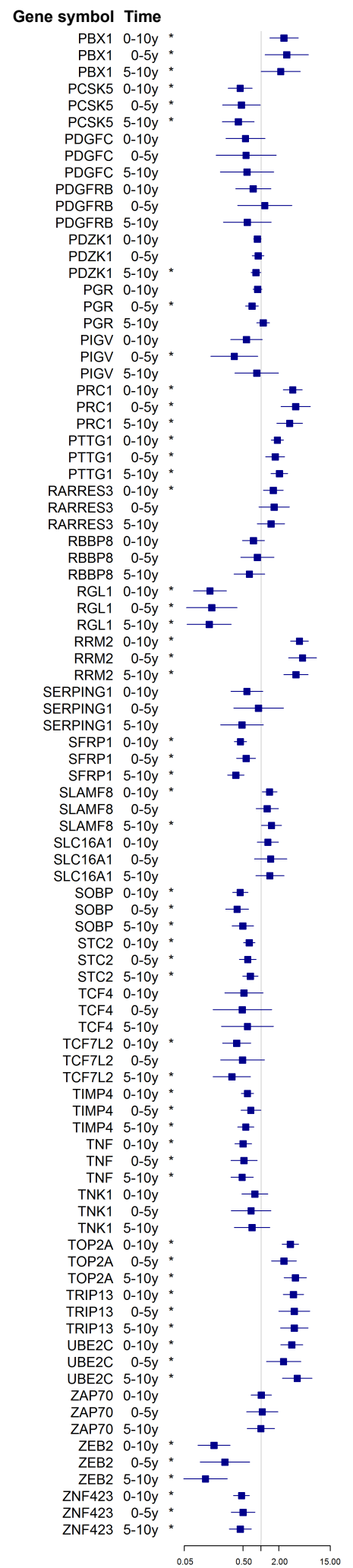

Supplement: Supplementary file 3 — Figure S1. Forest plot of HRs and CIs for the 92 genes assessed in TransATAC in univariate analyses. Asterisk denotes significance. (PDF 1497 kb) [file 13058_2018_1040_MOESM3_ESM.pdf]
