# Supplementary material for: Novel 18-gene signature for predicting relapse in ER-positive, HER2-negative breast cancer
Source: Breast Cancer Res. 2018 Sep 4;20:103. doi: 10.1186/s13058-018-1040-9 (PMC6122470; doi:10.1186/s13058-018-1040-9)
Supplement: Supplementary file 4 — Table S10. Likelihood ratio (LR) χ2 and p values for CTS and 10-year signature in three groups of POLAR validation set for 0–5 and 5–10 years of follow-up. Both univariate and multivariable analyses are presented for years 0–10, years 0–5, and years 5–10 separately. LR test based on Cox proportional hazards models for univariate and multivariable analyses. Differences in LR values (ΔLRχ2) were used. CTS was used as a covariate in the multivariable regressions. POLAR Molecular Predictors Of early versus LAte Recurrence in ER-positive breast cancer, CTS Clinical Treatment Score. (DOCX 15 kb) [file 13058_2018_1040_MOESM4_ESM.docx]

**Table S10. LRχ² and p-values for CTS and 10-year signature in three groups of POLAR validation set for 0-5 and 5-10 years of follow-up.**

|  | | | **All POLAR patients** | **Chemotherapy-treated** | **Chemotherapy-naïve** |
| --- | --- | --- | --- | --- | --- |
| 0-5 years | | | | | |
| No. of patients (relapses) | | | n=308 (133) | n=128 (57) | n=180 (76) |
| Univariate | CTS | LRχ^2^ | 22.09 | 15.25 | 10.08 |
|  |  | P | <0.001 | <0.001 | 0.002 |
|  | 10-year signature | LRχ^2^ | 14.59 | 7.41 | 7.26 |
|  |  | P | <0.001 | 0.007 | 0.007 |
| Multivariable comparison | CTS+10-year signature vs CTS | ΔLRχ^2^ | 7.59 | 5.95 | 2.72 |
|  |  | P | 0.006 | 0.015 | 0.099 |
| 5-10 years | | | | | |
| No. of patients (relapses) | | | n=285 (114) | n=113 (42) | n=172 (72) |
| Univariate | CTS | LRχ^2^ | 0.90 | 0.11 | 2.19 |
|  |  | P | 0.34 | 0.744 | 0.139 |
|  | 10-year signature | LRχ^2^ | 4.18 | 3.15 | 1.70 |
|  |  | P | 0.041 | 0.08 | 0.19 |
| Multivariable comparison | CTS+10-year signature vs CTS | ΔLRχ^2^ | 3.58 | 3.12 | 0.82 |
|  |  | P | 0.059 | 0.077 | 0.365 |

Both univariate and multivariable analyses are presented for years 0 to 10, years 0 to 5, and years 5 to 10 separately. Likelihood ratio test based on Cox proportional hazard models for univariate and multivariable analyses. Differences in likelihood ratio values (ΔLRχ^2^) were used. CTS was used as a covariate in the multivariable regressions. POLAR = molecular Predictors Of early versus LAte Recurrence in ER-positive breast cancer; CTS = clinical treatment score; LR = likelihood ratio.
